# Obesogenic Inflammatory Memory: A New Concept Related to the Dangerous Effects of Weight Cycling

**DOI:** 10.3390/biom16020193

**Published:** 2026-01-27

**Authors:** María del Carmen Navarro, María Dolores Hinchado, Elena Bote, Isabel Gálvez, Eduardo Otero, Miguel Palomino-Segura, Leticia Martín-Cordero, Eduardo Ortega

**Affiliations:** 1 Immunophysiology Research Group, Instituto Universitario de Investigación Biosanitaria de Extremadura (INUBE), 06006 Badajoz, Spain; cnavarropz@unex.es (M.d.C.N.); mhinsan@unex.es (M.D.H.); elenabote84@hotmail.com (E.B.); igalvez@unex.es (I.G.); eoteroc@unex.es (E.O.); miguelpalomino@unex.es (M.P.-S.); leticiamartin@unex.es (L.M.-C.); 2 Immunophysiology Research Group, Physiology Department, Faculty of Sciences, University of Extremadura, 06006 Badajoz, Spain; 3 Immunophysiology Research Group, Nursing Department, Faculty of Medicine and Health Sciences, University of Extremadura, 06006 Badajoz, Spain; 4 Immunophysiology Research Group, Nursing Department, University Center of Plasencia, University of Extremadura, 10600 Plasencia, Spain

**Keywords:** obesity, yo-yo dieting, anxiety-like behavior, sensorimotor capacity, inflammation, macrophage infiltration, adipose tissue, peritoneal macrophages, crown-like structures, M1 M2 macrophages

## Abstract

Obesity is associated with profound metabolic, inflammatory, and neurobehavioral dysfunctions. Dietary interventions leading to weight loss are commonly employed, yet it remains unclear whether all obesity-related alterations are fully reversed upon reaching normal body weight. Poor adherence to dietary regimens often results in weight cycling, or yo-yo dieting, characterized by repeated episodes of weight gain and loss, a phenomenon linked to adverse health outcomes. Here, we investigated the consequences of weight cycling in C57BL/6J mice. The Control Group was maintained on a standard chow diet throughout the protocol, whereas the experimental group underwent two alternating cycles of high-fat diet feeding (weight gain) and standard diet reversion (weight loss), until the end of the protocol where both groups reached 80 weeks of age. Despite achieving a final body weight and glucose and lipid metabolic profile comparable to lean controls, weight-cycled mice exhibited impaired sensorimotor function, increased anxiety-like behavior (evaluated through behavioral tests), and persistent inflammation, including a peritoneal macrophage pro-inflammatory profile and adipose tissue infiltration. We define this phenomenon as “obesogenic inflammatory memory”, highlighting that obesity leaves an immunological imprint that sustains inflammation even after normalization of weight and metabolic parameters. These findings demonstrate that weight cycling is associated with chronic macrophage-mediated inflammatory states, linked to long-term behavioral and neurological manifestations, and opening new avenues for future investigation and therapeutic approaches.

## 1. Introduction

According to the World Health Organization (WHO), obesity is defined as an abnormal or excessive accumulation of fat that may impair health [1]. It is considered one of the major global challenges due to its profound impact on population health worldwide. It is associated with comorbidities and metabolic dysfunctions, such as insulin resistance, which increase the incidence of severe diseases including atherosclerosis, type 2 diabetes, cardiovascular disease, infectious and autoimmune disorders, and cancer [2,3]. The global prevalence of obesity has nearly tripled since 1975 [1], and data from Europe in 2022 reported that 60% of the population is either overweight or obese [1].

A distinctive feature of this condition is the presence of elevated systemic levels of inflammatory molecules, which has long led obesity to be recognized as a chronic low-grade inflammatory disease [4,5,6,7]. Importantly, obesity also drives local immune-cell infiltration into metabolic organs, so systemic inflammation is paralleled by tissue-level immune activation. This chronic inflammatory state provides a biological link between obesity and many of its associated metabolic complications, as this inflammatory state is evident both systemically and locally, through macrophage infiltration in adipose tissue and pro-inflammatory activation in sites such as the peritoneum [8,9].

In addition, behavioral changes are thought to be linked to neurobiological mechanisms involving inflammatory pathways in both the central and peripheral nervous systems [4,10], showing that obesity is not only manifested at systemic or molecular levels but is also reflected in behavior. In fact, high-fat and cholesterol-enriched diets have been associated with increased aggression, anxiety, and depression, as well as with reduced exploratory behavior in novel environments [11,12,13,14]. Moreover, high-fat diets have also been reported to impair fundamental cognitive functions, leading not only to psychoneurological alterations but also to deficits in motor, sensorimotor, and exploratory activity in animal models. These effects are most likely due to the impact of diet on the hippocampus and prefrontal cortex, which are critical for cognitive performance [9,15].

Obesity is also characterized by a reduced innate immune capacity against pathogens [16,17], and increased susceptibility to infections, particularly viral infections, as has been observed in recent years with COVID-19 [18,19]. Optimal nutritional and metabolic homeostasis are crucial for maintaining proper immune function and overall health. Therefore, when a chronic imbalance between energy intake and expenditure occurs, leading to obesity, it is evident that both innate and adaptive immune responses are also impaired [20].

Weight loss is the primary and most critical goal for the management of obesity and its related comorbidities. Several intervention strategies have been proposed to achieve weight reduction, including anti-obesity drugs, bariatric surgery, and lifestyle modifications [21]. Among lifestyle interventions, one of the most widely accepted methods to promote weight loss is caloric restriction (either through reduced energy intake or the avoidance of high-fat diets). Weight loss achieved through a low-fat diet is considered a plausible, non-invasive, easy-to-follow strategy with numerous beneficial effects in obesity. However, despite their efficacy in the short term, long-term adherence is notoriously poor. Unfortunately, around 80% of individuals who successfully lose weight through dietary restriction eventually regain it within the following year [22,23]. Consequently, many individuals who are prone to weight gain follow diets for a limited period and then discontinue them, a phenomenon known as weight cycling or yo-yo dieting [24]. This phenomenon is characterized by repeated cycles of weight loss and regain, which may impact metabolic, cellular, and systemic functions, as well as significantly influence individual behavior [25].

Although obesity is a recognized risk factor for multiple chronic diseases, it remains unclear whether weight cycling itself may represent an even more detrimental component. In general terms, most concerns regarding the effects of weight cycling are related to morbidity and mortality, metabolism, and underlying inflammation. Research on this topic is still ongoing; however, many of the effects of this phenomenon at a general level remain unknown. The lack of a standard definition of weight cycling, the complexity of its patterns, and limitations in study design and analytical methods make it difficult to interpret results across studies [22]. It is particularly important to note that most weight cycling research has focused on the weight-gain phases, whereas the periods of weight loss or the subsequent lean state have received far less attention.

It has been demonstrated that weight cycling makes weight loss more difficult and weight regain easier, ultimately resulting in a progressive increase in body fat across successive weight cycles [26,27,28,29,30]. For instance, Brownell demonstrated that during a second weight loss–regain cycle, it took twice as long to lose the same amount of weight initially lost, and half the time to regain the same amount of weight gained during the first cycle [27]. Within this context, the term “obesogenic memory”, first introduced by Schmitz and colleagues [31], describes the notion that the body may have a “memory” or predisposition to regain weight after weight loss. This implies that the body “remembers” its prior obese state and tends to resist efforts to maintain sustained weight loss. While originally defined in metabolic terms, accumulating evidence indicates that this memory may also extend to immune and inflammatory pathways. Although the mechanisms generating inflammation in adipose tissue during obesity are relatively well understood, little is known about how adipose tissue leukocytes respond to weight cycling [32]. At the onset of weight loss, decreases in these inflammatory biomarkers can occur, suggesting a potential reduction in inflammation. However, during weight regain, these levels rise again, suggesting that weight cycling may sustain a fluctuating inflammatory state [33,34]. Importantly, most available studies focus on the inflammatory response during weight gain or during the early stages of weight loss, whereas virtually no research has examined the inflammatory status of animals that have already returned to a lean phenotype after undergoing one or multiple cycles of weight gain and loss. This represents a critical gap in the field, particularly given the potential implications for psychoneuroimmunoendocrine regulation. Understanding whether inflammatory alterations persist independently of current adiposity is therefore essential for interpreting the long-term biological impact of weight cycling and it is crucial for designing strategies aimed at preventing obesity-associated inflammation and improving weight maintenance.

In a previous work, our group established a high-fat diet-induced obesity model that revealed profound immunoneuroendocrine dysregulation, including alterations in stress-response biomarkers, anxiety-like behavior, and macrophage-mediated inflammatory dysfunction, thus validating the interdependence between metabolic, immune, and neuroendocrine systems in obesity [9]. The present study aimed to investigate the effects of weight cycling on the immunoneuroendocrine, metabolic, and behavioral parameters in formerly obese mice, and to compare this effect with their respective age-matched always-lean controls. Specifically, the objective of this study was to analyze the impact of two alternating cycles of high-fat diet feeding and subsequent reversion to a standard diet leading to weight loss, mimicking a weight cycling-like pattern, on metabolic profile, sensorimotor performance and anxiety-like behavior, together with the analysis of the inflammatory phenotype and innate response of peritoneal macrophages and macrophage infiltration in white adipose tissue (WAT). To the best of our knowledge, this is the first study to comprehensively integrate metabolic, immune, and behavioral assessments within a weight cycling model, providing an integrative perspective on the long-term consequences of repeated weight fluctuation, all of them evaluated in the weight-loss period (lean animals).

## 2. Materials and Methods

### 2.1. Experimental Design

A total of 12 male and female C57BL/6J mice (8 weeks old at the beginning of the experiment) were obtained from the Animal Facility of the University of Extremadura (Badajoz, Spain). Animals were individually housed under controlled environmental conditions (22–24 °C, 50–60% humidity) with a 12 h light/12 h dark cycle (lights on at 11:00 a.m.) and had ad libitum access to water and the assigned diet.

At the beginning of the experiment, animals were randomly divided into the following two groups:

- Control Group (n = 5, 2 males and 3 females): maintained on a standard chow diet (SD, SAFE A04, Augy, France) throughout the entire study.
- Weight Cycling Group (n = 7, 3 males and 4 females): subjected to two consecutive diet cycles, each consisting of a high-fat diet (HFD; SAFE 260HF, Augy, France) period (weight-gain phase) followed by a standard diet (SD; SAFE A04, Augy, France) period (weight-loss phase).

**Figure 1:**
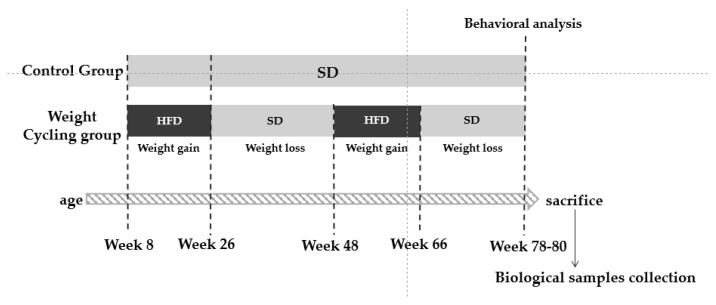
Experimental design. SD: Standard Diet; HFD: High Fat Diet.

At the start of the protocol, the Weight Cycling Group was fed the HFD for 18 weeks (until approximately 26 weeks of age), inducing obesity. Then, the HFD was replaced with the SD for 12 weeks, marking the first weight-loss phase, during which mice reached body weights similar to those of the Control Group. Animals remained on the SD for an additional 10 weeks to stabilize weight (total of 22 weeks of standard diet). Subsequently, they were re-exposed to the HFD for another 18 weeks (second weight-gain phase), leading to renewed obesity, and finally returned to the SD for 12 weeks (second weight-loss phase) until the end of the experiment when all animals were 78–80 weeks old. In parallel, the Control Group was maintained on a SD throughout the entire experimental period, remaining lean until the end of the study (78–80 weeks of age) (Figure 1).

Body weight and food intake were recorded weekly throughout the protocol. At the end of the experimental period, animals were anesthetized, and biological samples (blood, white adipose tissue, and peritoneal cells) were collected for further analysis.

All procedures were carried out in accordance with the European Directive 2010/63/EU and Spanish legislation for the protection of animals used in research. The study was approved by the Bioethics Committee for Animal Experimentation of the University of Extremadura (protocol number 70/2018, project IB18011).

### 2.2. Diet Protocol

The diet protocol was designed to induce and subsequently reverse obesity through alternating periods of high-fat and standard diet feeding, thereby mimicking a weight cycling (yo-yo dieting) pattern.

Mice in the Control Group received a standard chow diet (SD, SAFE A04, Augy, France) throughout the entire experimental period. This diet contains approximately 5% lipids, providing about 8% of total energy intake from fat.

In contrast, the Weight Cycling Group followed a sequence of high-fat diet (HFD; SAFE 260HF, Augy, France) and SD feeding phases across their lifespan. The HFD contains 36% fat, providing 58.8% of total energy intake from lipids, and is widely used to induce obesity and metabolic alterations in murine models [8]. Diet composition and energy content for both feeds are referenced in Table 1.

Before the administration of the HFD, during the first week of intake, a mixture of both diets (50% standard feed and 50% high-fat feed) was provided to prevent potential gastric damage due to a sudden diet change. A total of 40 g of standard feed was provided to lean animals weekly, while 30 g of high-fat feed was given per week to obese animals. Weekly intake and body weight were monitored for all mice.

### 2.3. Physical Condition, Sensorimotor, and Behavioral Tests

Behavioral tests were performed to evaluate physical condition, sensorimotor performance, and anxiety-like behavior in both experimental groups the day before the sacrifice. All tests were conducted during the animals’ active period (dark phase, 11:00–23:00 h) and under controlled temperature and lighting conditions (20 W red light). Mice were handled daily during the week preceding the behavioral assessments to minimize handling-related stress.

#### 2.3.1. Tests Assessing Physical Condition

##### Grip Strength Test

The grip strength test (BIO-GS3, Bioseb, Chaville, France) was used to measure grip strength followed by the procedure outlined by Cabe and colleagues [35].

For the performance of the test, mice were gently allowed to grasp a horizontal metal grid with its front paws. Then, the animal was placed in the horizontal plane, parallel to the sensor, pulling it backward by the tail. The force applied just before losing grip was recorded as the maximum tension. The test was performed in triplicate, and the result was calculated as the mean of the three measurements divided by the weight of the animal. The final result was referred to as the force value.

##### Tightrope Test

The apparatus consisted of a 60 cm long hemp rope, divided into segments of 10 cm and elevated to a height of 40 cm, positioned horizontally over a bed of shavings. The test began by placing the animal in the center of the tightrope. The maximal testing time was 30 s and was performed only once per individual.

The first aim of this test was to assess neuromuscular vigor. For this purpose, we focused on the parameter “percentage of falls”. Additionally, this test was also used to evaluate the coordination of the animals as a sensorimotor response, for which the parameter “percentage of animals that complete the test” was analyzed.

#### 2.3.2. Test Assessing Sensorimotor Response

##### Wire Rod Test

Following the protocol outlined by Baeza and colleagues [36], a wire rod (1 cm of diameter, 50 cm of length) divided into segments, suspended 30 cm above a soft bedding surface and supported by two platforms at the ends was used to perform the test. Each mouse was placed carefully in the center of the rod and the test concluded after 20 s or/if the animal managed to reach one of the ends. The test was conducted in duplicate. The parameter “percentage of mice that complete the test” evaluated the balance and “number of segments” was used to evaluate the coordination of the animals.

#### 2.3.3. Tests Assessing Anxiety-like Behavior

##### Elevated Plus Maze Test

The elevated plus maze (EPM) test was used to evaluate anxiety-like behavior in mice, exploiting their natural aversion to elevated and open areas. The apparatus consisted of two types of arms: two open arms without walls, allowing the animal to perceive the height and external environment, and two closed arms with high opaque walls, providing an enclosed and protected space. The time and frequency the mouse enters and spends on each arm determine the anxiety-like behavior of the animal [37,38].

The apparatus consisted of two open arms (without walls) measuring 50 cm in length and 10 cm in width, facing each other, and two closed arms (with walls 40 cm in height) of the same dimensions as the open arms, positioned perpendicular to the latter. It was constructed of rigid black plastic material and was elevated 62 cm above the ground on a metal support with four legs.

Testing was carried out under low-intensity red light (20 W) in a quiet environment. Each mouse was gently placed in the central area of the maze, facing a closed arm, and its activity was recorded for 5 min. The parameters used to evaluate the anxiety-like behavior of the animals were the number of entries in each of the arms and the time and percentage of time spent in them. This last parameter was calculated by dividing the time spent in each of the arms (in seconds) by the total duration of the test (300 s) and multiplying by 100. Increased exploration and time spent in the open arms are interpreted as reduced anxiety-like behavior, whereas a preference for the closed arms indicates higher anxiety levels.

##### Hole-Board Test

The hole-board test was used as a complementary measure of anxiety-like responses, based on the natural tendency of rodents to investigate novel environments. The procedure followed the general design described by Viveros and colleagues [39], with minor adaptations.

The apparatus consisted of an uncovered square platform (60 × 60 cm) with matte black walls (45 cm high) and a base divided into 36 equal squares (10 × 10 cm each) outlined in white. In the central region, four equispaced holes (3.8 cm in diameter) were incorporated, each filled with a contrasting red circular background.

Each animal was placed in one of the corners at the start of the test and allowed freely explore for 5 min under red light conditions, while its behavior was continuously recorded via video. The anxiety-like behavior was inferred from the head-dipping activity, defined as the action of inserting the head into one of the holes. Two parameters were analyzed: the number of head dips and the total duration of head-dipping behavior (in seconds). According to File and Wardill [40], the frequency and duration of head dips are inversely proportional to anxiety levels.

### 2.4. Body Measurements and Collection of Biological Samples

Body weight was monitored weekly throughout the duration of the experimental protocol. Prior to sample collection on the day of sacrifice, animals underwent a 12 h fasting period. Mice were anesthetized with isoflurane (initial dose: 3–5%; maintenance: 1.5–3%) administered via inhalation, following standard procedures to minimize stress and ensure rapid induction.

Biological samples were collected from live, anesthetized animals. Whole blood was obtained by cardiac puncture, and samples were immediately processed to assess fasting blood glucose and lipid profile, including total cholesterol, high-density lipoprotein cholesterol (HDL-C), calculated low-density lipoprotein cholesterol (cLDL-C), and triglycerides (TG), using a portable analyzer (LUX^®^, Microcaya, Bilbao, Spain).

Peritoneal cells were collected by injecting 4 mL of sterile phosphate-buffered saline (PBS) medium into the peritoneal cavity, followed by gentle massage and aspiration of the peritoneal exudate, which was transferred to polypropylene tubes until further processing.

WAT was carefully dissected through laparotomy, immediately embedded in an optimal cutting temperature (OCT) compound, snap-frozen in liquid nitrogen, and stored at −80 °C until histological and immunofluorescence analyses.

### 2.5. Assessment of Phagocytic and Oxidative Burst, and the Inflammatory Profile of Peritoneal Macrophages

For the assessment of phagocytic and microbicidal capacity in the peritoneal exudate, flow cytometry analysis was performed following a modification of the technique described by Robinson and Carter [41]. Using this technique, the capacity of macrophages to engulf bacteria and generate superoxide anion (O_2_-, indicative of oxygen-dependent microbicidal activity) can be accurately determined by the mean fluorescence intensity (MFI) of actively phagocytic cells, as measured by hydroethidine (HE, a specific test to detect intracellular superoxide anion production by NADPH oxidase).

First, *Escherichia coli* (*E. coli*) was obtained at an optical density of 1.6 (O.D.), fixed in paraformaldehyde (PFA) (1%) and washed and filtered (0.22 μm diameter filter) with PBS. Subsequently, the bacteria were stained with FITC (F3651) (fluorescein isothiocyanate) (Sigma-Aldrich Merck KGAa, Darmstadt, Germany) at a final concentration of 30 μg/mL for 30 min at 37 °C in darkness and agitation and then washed twice with PBS. Once stained, the bacteria were opsonized by incubating them with human serum for 3 h at 37 °C in darkness and agitation and then resuspended in PBS with 2% fetal bovine serum (FBS) (A5256801) (Thermo Fisher Scientific, Waltham, MA, USA).

Next, 100 μL of peritoneal exudate from each mouse were incubated for 30 min at 37 °C in darkness and agitation with: 25 μL of opsonized *E. coli*-FITC bacteria, Hoechst 33342 (H1399) (Thermo Fisher Scientific, Waltham, MA, USA) (10 μg/mL), Topro-3 iodide (T3605) (Thermo Fisher Scientific, Waltham, MA, USA) (0.1 µM), and 375 µL of PBS 1X in 2% de FBS. After 30 min of incubation, hydroethidine (HE) was added, completing the incubation for another 30 min. A control was performed using 50 μL of peritoneal exudate along with Hoechst 33342 (10 μg/mL), Topro-3 iodide (0.1 µM), and 450 µL of PBS 1X in 2% de FBS. Finally, the sample was analyzed by flow cytometry (MACSQuant VYB, Miltenyi Biotec, Barcelona, Spain) with 3 lasers (405 nm, 488 nm, 561 nm), and the results were processed and analyzed using the “FlowJo v10” software. Results were expressed as phagocytic and microbicidal percentages (percentage of cells that have ingested bacteria and percentage of cells that have produced superoxide anion to destroy them, respectively) as well as by their phagocytic and microbicidal activity (expressed by MFI, shown by the phagocytic cells when ingesting the bacteria and by the MFI exhibited by the production of superoxide anion indicating the amount of bacteria ingested by cells, respectively).

For the evaluation of the inflammatory profile of peritoneal macrophages and their activity, the membrane expression of CD11c and CD206, as well as the intracellular expression of iNOS and ARG-1 were assessed. Aliquots of 200 μL of cell suspension (1 × 10^7^ cells/mL) obtained from peritoneal lavage were used. After resuspension in PBS and 0.5% BSA and 2 mM EDTA together with Inside Fix (Miltenyi Biotec, Madrid, Spain), the samples were incubated at 25 °C in the dark with agitation and centrifugated and resuspended again in PBS, 0.5% BSA, and EDTA. For antibody staining the samples were resuspended in Inside Perm (Miltenyi Biotec, Madrid, Spain) and incubated with CD11c-PE, CD206-FITC, iNOS-Alexa430, and ARG1-PE antibodies. Antibody concentrations were determined by titration. Subsequently, they were washed and resuspended again in Inside Perm and analyzed in the Cytoflex cytometer (Beckman Coulter, Brea, CA, USA) and the data were processed using CytExpert v2.6 software.

### 2.6. Assessment of Macrophage Infiltration and Adipocyte Size in WAT

WAT was cut into 12–15 µm sections in a cryostat (LEICA, CM 1950, Leica Biosystems, Lake, IL, USA) at −30 °C and mounted on Superfrost^®^ Plus microscope slides (Thermo Fisher Scientific, Braunschweig, Germany). Samples were stored at −20 °C until the immunostaining procedure. A hydrophobic barrier pen (ImmEdge Hydropho-bic Barrier PAP Pen H-4000, Vector Laboratories, Newark, CA, USA) was used to encircle the sections before staining.

For the evaluation of adipocyte size in adipose tissue, hematoxylin–eosin and Oil red O staining was performed on previously processed and fixed adipose tissue sections for subsequent visualization using a conventional optical microscope (Axioskop model, Zeiss, Oberkochen, Germany). The AxionVision LE visualization software (Carl Zeiss™, Madrid, Spain) was utilized for observing adipocytes at 40× magnification and measuring them. To determine size, the diameter of all observable adipocytes per field was calculated across 10 randomly selected fields per mouse. Conversion of diameters from pixels to micrometers was conducted.

For the immunofluorescence technique, slides containing the adipose tissue samples were washed with PBS + Triton X-100 (Merck KGAa, Darmstadt, Germany) and then fixed with 4% PFA for 5 min. After a series of washes with PBS + Triton X-100 and with PBS + gelatin (PanReac AppliChem, Barcelona, Spain) + Triton X-100, non-specific binding sites were blocked with PBS + gelatin + Triton X-100 + lysine (Merck KGAa, Darmstadt, Germany) for 1 h. Finally, F4/80 (Alexa Fluor^®^ 488 Anti-mouse F4/80 Antibody, BioLegend, San Diego, CA, USA) antibody was added at concentrations determined after titration. The samples were then incubated overnight in a humid chamber in darkness. The next day, slides were washed again with PBS + Triton X-100 and with PBS + gelatin + Triton X-100. DAPI 2 μM (PanReac AppliChem, Barcelona, Spain) was added as a nuclear stain. After 15 min of incubation, the slides were washed with PBS. Coverslips and Mowiol^®^ 40-88 (Sigma-Aldrich, Darmstadt, Germany) were used for mounting the samples. The samples were stored at 4 °C in a humid chamber in darkness until they were visualized under the fluorescence microscope for the counting of infiltrated macrophages in the adipose tissue.

Immunostained adipose tissue sections were observed under a conventional transmitted light and fluorescence microscope (Nikon ECLIPSE 80i, Nikon, Tokyo, Japan), obtaining digital images with a camera attached to the microscope (Nikon Digital Camera DXM 1200F, Nikon, Tokyo, Japan). The images were overlaid and optimized using Adobe PhotoShop v.CS4 software (Adobe, San Jose, CA, USA). For cell counting, 10 randomly chosen fields of view were selected using a 40× objective and the number of F4/80+ cells (considered macrophages), both solitary and aggregated, forming the so-called “crown-like structures” (CLS) was counted using ImageJ imaging 1.54p software.

### 2.7. Statistical Analysis

The mean ± standard error of the mean (SEM) was used to express values, with the normal distribution of variables confirmed by the Kolmogorov–Smirnov test. Student’s *t*-test was used to compare pairs of groups, whether the samples were paired or non-paired. Statistical significance was determined by the probability value “*p*” (*p*-value), with thresholds set at *p* < 0.05, *p* < 0.01, and *p* < 0.001, each indicating increasing levels of significance. Effect size (ES) was calculated using Cohen’s d and interpreted as very low (d < 0.2), low (0.2 < d < 0.5), medium (0.5 < d < 0.8), and high (d > 0.8). IBM SPSS Statistics v31 and Graphpad Prism 8.0.1 have been used for data analysis.

## 3. Results

### 3.1. Body and Metabolic Parameters

The body and metabolic parameters regarding control and weight-cycled animals are set out in Table 2. Body weight, fasting glucose, cholesterol, and triglycerides systemic concentrations were similar between the two experimental groups. The only difference observed was a lower daily feed intake in the Weight Cycling Group which is expected since the values represent the average consumption across the entire protocol (including both fattening and weight-loss phases) and reflect the satiety induced by the hyperlipidic content of the chow administered, as already demonstrated in previous studies [9].

**Figure 2:**
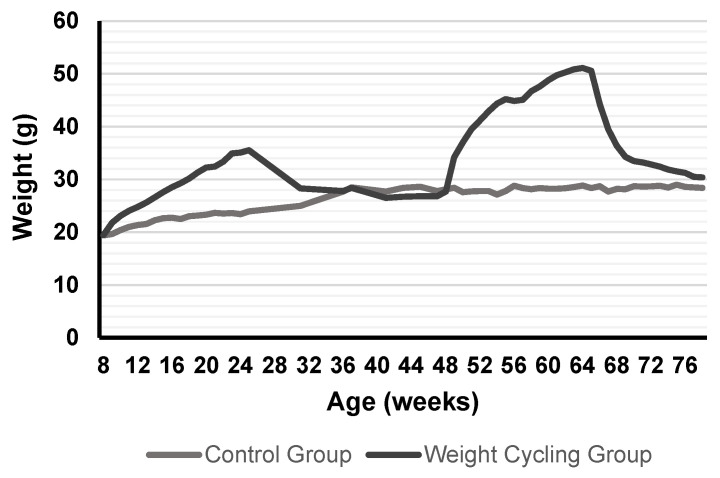
Weight evolution in mice from Control Group and Weight Cycling Group for 70 weeks. Each value in the graph corresponds to the average of the weights recorded weekly from the start of the protocol (8–10 weeks of age) until the day of sacrifice (approximately 80 weeks of age). Values expressed in grams.

**Figure 3:**
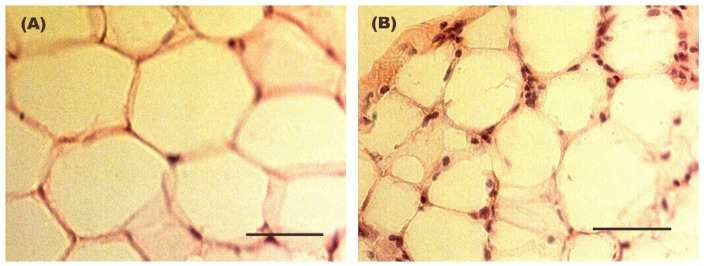
Representative hematoxylin–eosin-stained images of adipocyte size in WAT from (**A**) a Control Group mouse and (**B**) a Weight Cycling Group mouse. Mean adipocyte diameter was 70.5 ± 3.4 µm in the Control Group and 63.7 ± 3.0 µm in the Weight Cycling Group. In addition to adipocytes nuclei, the stained sections show multiple discrete nuclei corresponding predominantly to infiltrating leukocytes. These images illustrate a reduction in adipocyte size while showing a persistence of inflammatory nuclei in the adipose tissue under weight cycling conditions. Scale bar: 50 µm.

Figure 2 shows the evolution of body weight during the entire experimental protocol. Figure 3 illustrates the size of the adipocytes from both Control and Weight Cycling Groups. There were no differences found between them, which seems to corroborate that these animals, in addition to restoring weight after diet cycles, also restore the size of their adipocytes, probably due to the loss of lipid reserves.

### 3.2. Behavioral Tests

#### 3.2.1. Physical Condition

**Figure 4:**
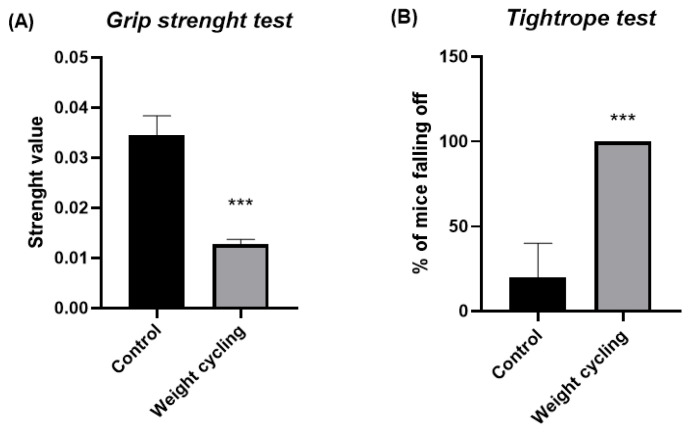
Physical condition assessment in Control and Weight Cycling Groups. (**A**) Grip strength test. (**B**) Tightrope test. Each column represents the mean ± SEM of the values determined in 5 animals from Control Group and 7 animals from Weight Cycling Group. *** *p* < 0.001, compared to the Control Group value (Student’s *t*-test). ES for grip strength test = 3.79; ES for tightrope test = 2.83.

The grip strength and the tightrope test were used to assess mice’s physical condition (Figure 4).

Mice subjected to repeated cycles of weight gain and loss (Weight Cycling Group) showed a significantly lower forelimb grip strength compared with lean controls (Control Group) (*p* < 0.001) (Figure 4A). This finding indicates that weight cycling negatively affects muscular strength and physical performance.

Similarly, in the tightrope test, a higher percentage of falls was observed in mice from the Weight Cycling Group compared with the Control Group (Figure 4B). These results confirm that repeated weight cycling leads to a pronounced decline in muscular vigor, suggesting impaired neuromuscular performance associated with the fluctuation between obesity and weight-loss periods.

#### 3.2.2. Sensorimotor Responses

**Figure 5:**
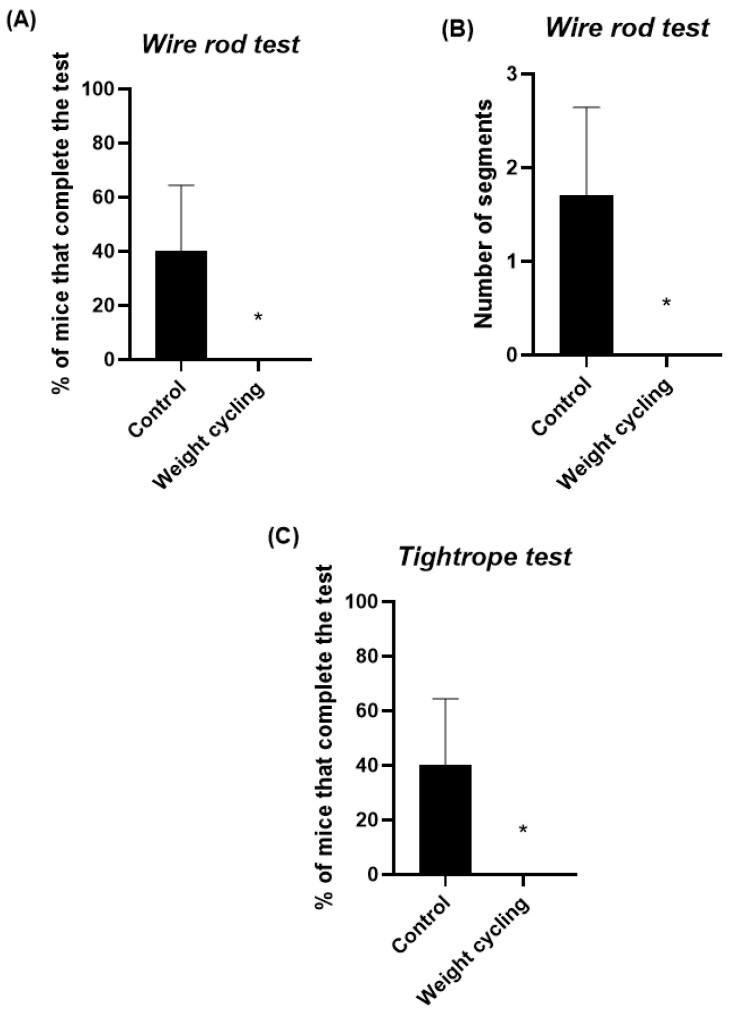
Assessment of sensorimotor performance in Control and Weight Cycling Groups. (**A**) Balance evaluation using the wire rod test. (**B**) Coordination assessment using the wire rod test. (**C**) Coordination evaluation through the tightrope test. Each column represents the mean ± SEM of the values determined in 5 animals from Control Group and 7 animals from Weight Cycling Group. * *p* < 0.05, compared to the Control Group value (Student’s *t*-test). ES for % of mice that complete the test in the wire rod test = 1.15; ES for number of segments = 1.27; ES for % of mice that complete the test in the tightrope test = 1.15.

Figure 5 shows the results of the parameters assessing balance and coordination, evaluated through the wire rod and the tightrope tests. In the wire rod test, the percentage of animals reaching the end of the structure was lower for the Weight Cycling Group (Figure 5A), indicating a reduced equilibrium performance in these animals. Similarly, in the wire rod test, mice from the Weight Cycling Group completed a significantly lower number of segments (*p* < 0.05) compared to the Control Group, indicating in this case impaired motor coordination (Figure 5B). Regarding the tightrope test, which also assessed motor coordination, none of the mice in the Weight Cycling Group were able to complete the test, whereas 40% of the Control Group successfully did so (*p* < 0.05), further confirming the presence of impaired motor coordination resulting from repeated cycles of weight gain and loss (Figure 5C). These findings further support that repeated weight cycling markedly compromises motor coordination and balance performance.

#### 3.2.3. Anxiety-like Behavior

**Figure 6:**
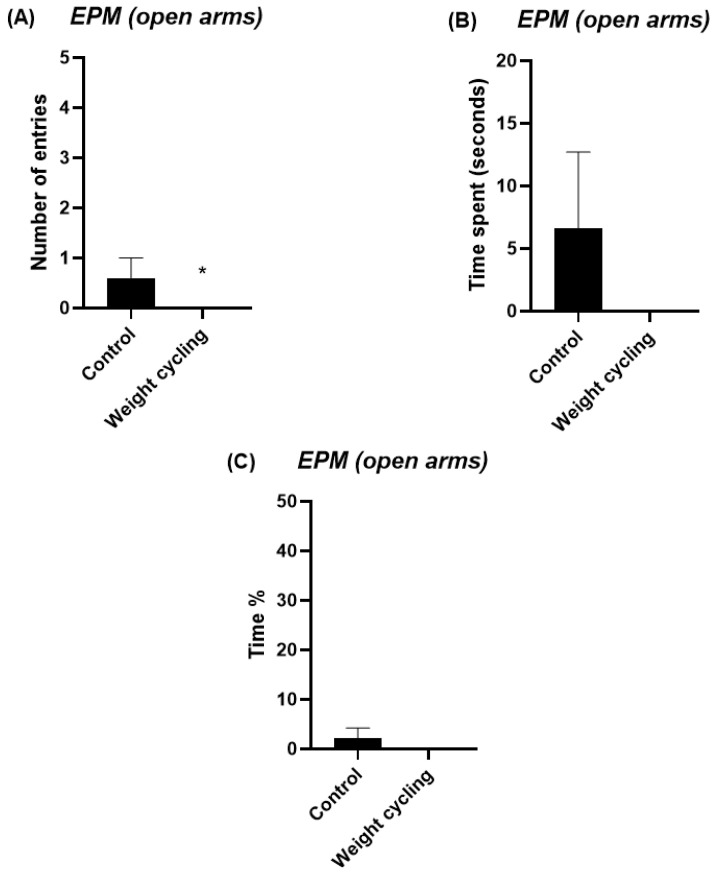
Assessment of anxiety-like behavior in Control and Weight Cycling Groups using the elevated plus maze test. (**A**) Number of entries into the open arms. (**B**) Time spent in the open arms (seconds). (**C**) Percentage of time spent in the open arms. Each column represents the mean ± SEM of the values determined in 5 animals from the Control Group and 7 animals from the Weight Cycling Group. * *p* < 0.05, compared to the Control Group value (Student’s *t*-test). ES for number of entries = 1.06; ES for time spent = 0.76; ES for time % = 0.76.

**Figure 7:**
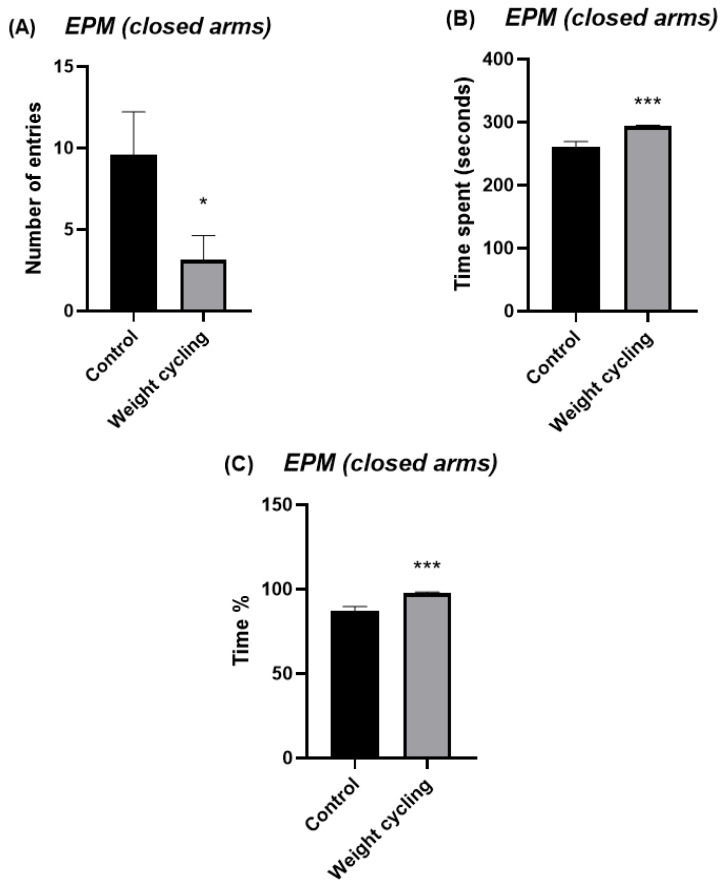
Assessment of anxiety-like behavior in Control and Weight Cycling Groups using the elevated plus maze test. (**A**) Number of entries into the closed arms. (**B**) Time spent in the closed arms (seconds). (**C**) Percentage of time spent in the closed arms. Each column represents the mean ± SEM of the values determined in 5 animals from the Control Group and 7 animals from the Weight Cycling Group. * *p* < 0.05, *** *p* < 0.001, compared to the Control Group value (Student’s *t*-test). ES for number of entries = 1.35; ES for time spent = 2.65; ES for time % = 2.65.

The EPM test was used to evaluate the anxiety-like behavior of mice. This test measures the number of entries and the time spent in both the open and closed arms of the structure and are commonly used as indicators of anxiety levels: a preference for closed arms is associated with increased anxiety, whereas greater exploration of the open arms reflects a lower anxiety-like state. The Weight Cycling Group exhibited a significant reduction in the number of entries into the open arms compared with the Control Group (*p* < 0.05) (Figure 6A) and tended to spend less time in these arms (*p* = 0.07) (Figure 6B,C). Regarding the closed arms, the Weight Cycling Group also made fewer entries (*p* < 0.05) (Figure 7A) but spent significantly more time in them compared with controls (*p* < 0.001) (Figure 7B,C), indicating an increased anxiety-like behavioral pattern in mice subjected to weight cycling.

**Figure 8:**
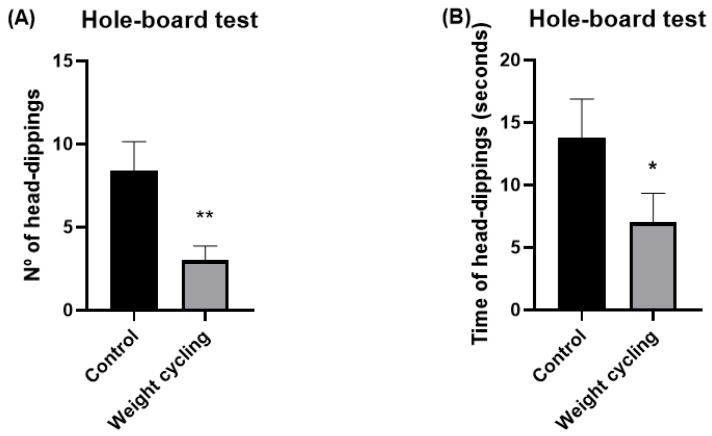
Assessment of anxiety-like behavior in Control and Weight Cycling Groups using the hole-board test. (**A**) Number of head-dippings. (**B**) Time spent performing head-dippings (seconds). Each column represents the mean ± SEM of the values determined in 5 animals from the Control Group and 7 animals from the Weight Cycling Group. * *p* < 0.05, ** *p* < 0.01, compared to the Control Group value (Student’s *t*-test). ES for number of head-dippings = 1.77; ES for time of head-dippings = 1.05.

In addition, the hole-board test was also used to further evaluate the anxiety-like behavior of the animals. In this test, mice are allowed to freely explore a board with evenly spaced holes, and their exploratory behavior is quantified by the number and duration of head-dipping events, cases where the animal lowers its head into the holes. These parameters are considered reliable indicators of emotional state, as both the frequency and duration of head-dippings are inversely related to anxiety levels [40]. As shown in Figure 8, both the number and time of head-dippings were significantly lower in the Weight Cycling Group compared with the Control Group (*p* < 0.01 and *p* < 0.05, respectively), confirming an enhanced anxiety-like behavioral profile in mice subjected to repeated weight gain and loss.

### 3.3. Phagocytic and Oxidative Burst

The following tables summarize the results of the phagocytic and microbicidal capacities of peritoneal macrophages from animals in each experimental group. No statistically significant differences were observed for any of the evaluated parameters, indicating that undergoing two alternating cycles of weight gain and loss did not impair either the phagocytic (Table 3) or the microbicidal capacity (Table 4) of peritoneal macrophages.

### 3.4. Inflammatory Profile of Peritoneal Macrophages

Regarding the inflammatory polarization of peritoneal macrophages, two main phenotypic profiles can be distinguished: the pro-inflammatory M1 subtype, characterized by the expression of CD11c and iNOS, and the anti-inflammatory M2 subtype, defined by the presence of CD206 and ARG-1. The following table displays the percentage of cells corresponding to each phenotype.

The results showed that the Weight Cycling Group exhibited a significantly higher percentage of CD11c^+^ macrophages (*p* < 0.05) (Table 5), a marker associated with the pro-inflammatory M1 phenotype. Thus, formerly obese mice that underwent repeated cycles of weight gain and loss displayed a more pro-inflammatory peritoneal macrophage profile compared to those that remained lean throughout the entire experimental protocol.

### 3.5. Macrophage Infiltration in WAT

The frequency and distribution of infiltrated macrophages was assessed in WAT using F4/80 antibody for detecting those cells.

**Figure 9:**
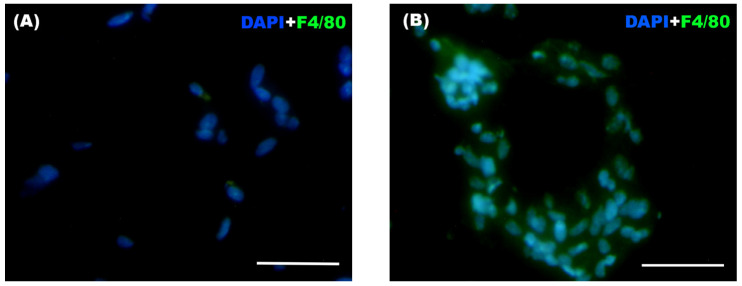
Representative immunofluorescence images of WAT from (**A**) a Control Group mouse and (**B**) a Weight Cycling Group mouse. Macrophages were identified by F4/80^+^ staining (green), and nuclei were counterstained with DAPI (blue) at 40× magnification, with 10 random fields photographed for each blinded sample. Scale bar: 50 µm.

Significant differences were observed between both groups in the total number of infiltrating macrophages and in the presence of crown-like structures (CLS) within the WAT (Table 6), with both parameters being markedly higher in the Weight Cycling Group compared to the Control Group. These differences can also be visually appreciated in Figure 9, where representative immunofluorescence images illustrate the higher macrophage infiltration and CLS formation in the Weight Cycling Group compared to the Control Group.

## 4. Discussion

Despite the inherent limitations in studying weight cycling, the complexity of its patterns, and the controversies surrounding its effects, it is evident that body weight fluctuations resulting from the withdrawal of a high-fat diet exert a significant impact on multiple physiological systems. Unlike the concept of obesogenic memory, which primarily refers to a predisposition to weight regain and metabolic dysfunction after weight loss [31], the present study experimentally demonstrates that repeated weight gain and loss can imprint a persistent macrophage-mediated inflammation even after weight normalization, providing evidence for the concept of an “obesogenic inflammatory memory”. It is important to note that aging is inherently associated with a progressive increase in low-grade inflammation as well as with declines in stress- and anxiety-related behaviors [42]. For this reason, we highlighted the inclusion of an age-matched Control Group in our experimental protocol, thus avoiding the potential interference of aging. This experimental design allowed us to discern the effects of weight cycling from those attributable to aging alone, concluding that the effects induced by weight cycling are either independent of, or exacerbate, those associated with aging. This model may contribute to a better understanding of how weight cycling through diet interventions influences long-term metabolic and immune homeostasis, opening new avenues for therapeutic approaches.

In our model, after two periods of withdrawal from a high-fat diet, the animals ultimately reached body weights comparable to those of the age-matched Control Group (which remained lean throughout the entire experimental protocol) with no significant differences in glucose and lipids metabolic parameters related to their age-matched lean counterparts. These findings are particularly relevant, as they indicate that although mice experienced substantial weight gain during specific phases of life, once their body weight returned to normal, their metabolic profile also reverted to levels comparable to those of mice that remained lean throughout the protocol. In agreement with our observations, many cross-sectional and prospective studies have reported no associations between weight cycling and altered glucose, total cholesterol, or HDL levels [43,44]. However, it is also important to note that the negative effects of weight cycling may vary depending on the duration and frequency of weight-loss and weight-regain phases, as well as on diet composition, the subject’s physical activity, and the specific phase of the cycle in which the assessment is performed (whether during obesity or after weight normalization such as the case of this study).

We have previously demonstrated that, in addition to increased levels of glucose, cholesterol, and triglycerides, our obese model showed marked impairments in physical and sensorimotor performance, together with pronounced anxiety-like behavior and an enhanced stress response [9]. Although normalization of body weight appears to restore metabolic parameters, the present findings reveal that physical condition does not fully recover after repeated cycles of weight gain and loss. Both the grip strength and tightrope tests demonstrated a significant decline in muscle strength and overall physical capacity in animals subjected to weight cycling, suggesting a deterioration of neuromuscular performance despite the restoration of a lean phenotype. This impairment could be associated with the chronic low-grade inflammatory environment characteristic of obesity, which affects muscle remodeling by reducing angiogenesis and myocyte formation while promoting fibrotic and adipose tissue deposition within skeletal muscle [45]. This structural remodeling is likely compounded by the disproportionate expansion of fat mass relative to lean mass during weight-gain phases, such that individuals undergoing diet cycling repeatedly lose and regain fat while progressively losing muscle, a process that is more difficult to reverse. Additionally, intramuscular lipid accumulation may impair amino acid uptake and protein synthesis in skeletal muscle [46], further contributing to sustained reductions in physical performance. Moreover, low-grade inflammatory environment may contribute to neuroinflammatory processes characterized by sustained activation of central immune cells, particularly microglia and astrocytes, potentially disrupting neuronal homeostasis and impairing neural circuits involved in motor control, a mechanism that may be driven by peripheral inflammatory mediators promoting central immune activation and neuronal dysfunction in brain regions involved in coordination and motor performance [47,48]. Altogether, and in agreement with prior evidence showing impaired physical and sensorimotor responses in obese mice relative to lean controls [9], our results suggest that the neuromuscular consequences of cyclical weight fluctuations persist even after the apparent recovery of metabolic homeostasis.

Consistent with the observed decline in muscle strength, our results also demonstrate that both balance and coordination were significantly impaired in the Weight Cycling Group, confirming a persistent negative impact on motor performance. It has been reported that excessive weight gain and increased circulating lipids can have detrimental effects on brain function [49,50], potentially disrupting central nervous system pathways involved in motor control, and recurrent changes in body composition due to alternating weight gain and loss may affect the balance between fat and muscle mass [51]. Thus, to the best of our knowledge, our findings provide the first experimental evidence that progressive cycles of weight gain and loss deteriorate sensorimotor responses, specifically balance and coordination, even after body weight normalization and recovery of metabolic parameters.

Notably, from a behavioral perspective, our findings also demonstrate that repeated cycles of weight gain and loss are associated with increased anxiety-like behavior, indicating that weight cycling may have a substantial impact on emotional regulation and stress reactivity. Previous human studies have already highlighted this connection, showing that recurrent dieting and subsequent weight regain are linked to higher levels of anxiety, depressive symptoms, and overall psychological distress [52,53,54,55]. These outcomes have often been attributed to the frustration and perceived failure associated with repeated weight loss attempts, as well as to sociocultural pressures surrounding body image and the maintenance of an “ideal” weight. In our study, an objective model mouse subjected to repeated high-fat diet cycles also displayed clear signs of anxiety-like behavior despite recovering a lean phenotype, showing the novelty of the present results. The persistence of anxiety-like features after body weight normalization suggests that weight cycling may trigger long-term neurobiological changes that outlast the metabolic alterations initially induced by obesity.

During obesity, the expansion of adipose tissue is accompanied by an increased recruitment and activation of immune cells, particularly macrophages, which contribute to the establishment of a chronic low-grade inflammatory state [8,9,56]. However, the extent to which this inflammatory response persists or is resolved after repeated weight fluctuations remains poorly understood, and nothing has been reported during the weight normalization period (lean state). In the present study, the normalization of adipocyte size after weight loss indicates a morphological recovery of adipose tissue; however, this does not necessarily imply a complete resolution of the inflammatory environment. Several mechanisms may contribute to this persistent inflammation despite reduced adipocyte size; for example, obesity is known to induce long-lasting remodeling of the extracellular matrix, including fibrosis and collagen deposition, which can persist after weight normalization and promote macrophage-driven inflammation [57] or even alterations in lipid composition within adipose tissue, rather than adipocyte size per se, may continue to provide inflammatory stimuli [58], including a persistent macrophage-mediated inflammation.

In this context, evaluating the impact of weight cycling on macrophage function provides valuable insight into the long-term consequences of alternating periods of obesity and weight normalization. In relation to the phagocytic and microbicide capacities of peritoneal macrophages, no significant differences were found between both groups. Although obesity has been shown to alter the metabolic environment and innate immune responses of peritoneal macrophages [9,16,17], it is plausible that these cells can recover their phagocytic and microbicidal functions once normal body weight and metabolic parameters are restored following high-fat diet withdrawal. This study represents, based on current knowledge, the first analysis of the phagocytic and microbicidal capacities of macrophages in animals subjected to repeated cycles of weight gain and loss, and suggests that, despite fluctuations in nutrient intake and body weight, lifelong cycles of weight gain and reduction do not appear to exert a lasting detrimental effect on the innate immune response of macrophages against pathogens once a lean state is reestablished, at least in our experimental model.

However, when evaluating inflammatory phenotype of peritoneal macrophages and macrophage infiltration in WAT, our results clearly show a persistent inflammation mediated by macrophages. Obesity is widely recognized as a state of chronic low-grade inflammation, characterized by the dysregulation and release of adipokines and pro-inflammatory cytokines and also by an increased proportion of pro-inflammatory macrophages together with enhanced macrophage infiltration into adipose tissue [8,9,59,60,61]. This inflammatory environment plays a central role in metabolic dysfunction, contributing to insulin resistance and tissue damage. Given this, it could be hypothesized that weight cycling might partially mitigate inflammatory damage through the weight-loss periods when animals return to the lean phenotype. However, several studies have shown that mere reductions in body mass are often insufficient to reverse the pro-inflammatory milieu established during obesity [20,25,33]. In line with this evidence, a significant increase in the percentage of peritoneal macrophages expressing CD11c (a marker associated with the pro-inflammatory M1 phenotype) was observed in our lean animals after weight cycling, probably without a complete polarization because it is not accompanied by increases in the other M1 markers (iNOS) or in the iNOS^+^/ARG-1^+^ ratios. In addition, our results also showed persistent increase in macrophage infiltration and CLS formation within the adipose tissue of the Weight Cycling Group, indicating the persistence of an active inflammatory process of macrophages. Thus, despite the restoration of normal adipocyte size, body weight, and a lean metabolic profile, the inflammatory environment characteristic of obesity was not completely resolved. These results allow us to introduce the concept of “obesogenic inflammatory memory”, referring to the persistent macrophage-mediated inflammation induced by obesity in lean individuals who have experienced weight cycling throughout adult life.

Interestingly and paradoxically, this sustained inflammation does not appear to translate into a significant worsening of metabolic parameters. These results are consistent with previous studies that also failed to find a clear relationship between weight cycling and glucose or lipid profiles [43,44]. Besides the increased proportion of pro-inflammatory peritoneal macrophages, the elevated macrophage infiltration and the presence of inflammatory “footprints” in adipose tissue, such as CLS, no major alterations in glucose or lipid metabolism were observed in the Weight Cycling Group.

The concept of “obesogenic memory” suggests that the organism retains a predisposition to regain weight after weight loss, maintaining a “trace” of its prior obese state [31] with epigenetic mechanisms appearing to be involved in this process [62,63]. Moreover, it has been proposed that this obesogenic memory may perpetuate metabolic and inflammatory disturbances in adipose tissue through inflammatory lymphocyte subsets, including Th1 and Th17 cells, in obese animals [64]. Supporting this notion, Blaszczak and colleagues [65] demonstrated that macrophages contribute to adipose tissue inflammation and insulin resistance during a single cycle of weight loss, highlighting the sensitivity of adipose immune cells to previous dietary exposures. Our results suggest that macrophage-mediated inflammation plays a central role in sustaining adipose tissue inflammation even in the context of diet-induced obesogenic memory with more than one single weight-loss cycle. This interpretation is conceptually consistent with the findings of Barbosa-da-Silva and colleagues [33], who reported that body weight reduction during weight cycling is insufficient to restore the elevated levels of adipokines and pro-inflammatory cytokines induced by high-fat feeding.

Therefore, the concept of “inflammatory obesogenic memory” is sustained by the macrophage-driven inflammatory state identified in this study, suggesting that obesity can induce long-lasting, possibly irreversible, inflammatory reprogramming in these cells. Epigenetic mechanisms, such as DNA methylation [62,63] might also contribute, at least partially, to the persistent inflammatory state of macrophages. Moreover, we may hypothesize that this persistent pro-inflammatory state, detected both in peritoneal and adipose tissue macrophages, could also extend to microglial cells in the brain. This mechanism might contribute to the sustained anxiety-like behavior and impaired coordination observed in our weight-cycled animals. Given the close immunoneuroendocrine relationship within the gut–brain axis, a pro-inflammatory state in the gastrointestinal tract, potentially mediated by microbiota alterations and exacerbated by yo-yo dieting, could promote microglial activation and represent a mechanistic link between weight cycling, anxiety, stress, and sensorimotor dysfunction [66,67]. Conversely, we cannot exclude a bidirectional relationship in which anxiety itself may contribute to gut dysbiosis, further promoting chronic inflammation in a vicious cycle. Indeed, as previously mentioned, recurrent weight fluctuations in humans have been associated with increased risk of depression, mental health disorders, and reduced psychological well-being [52,53,54,55]. The results presented here are, to our knowledge, the first to experimentally confirm these effects in an objective murine model, emphasizing the interplay between inflammatory response, anxiety-like behavior, and motor performance. These findings may also have potential translational clinical relevance in situations involving rapid weight loss induced by nutritional and exercise-based interventions [68], as well as in the context of personalized medicine by evaluating the results of both adherence and effectiveness of dietary interventions throughout life. Nevertheless, the present study opens new research approaches, and further studies are clearly needed to better understand the underlying immunophysiological mechanisms, particularly those related to immune-cell metabolic reprogramming, the gut–brain axis interaction linked to gut microbiota alterations, together with the potential epigenetic mechanisms associated with the persistent inflammation.

In addition, although no statistically significant sex-related differences were observed, a potential limitation of this study is the relatively small sample size, which may have limited statistical power for some biomarkers showing only strong trends. Nevertheless, in our opinion, this fact also reinforces the physiological relevance of parameters in which clear statistical differences were detected.

## 5. Conclusions

Our findings demonstrate that weight cycling induced by the withdrawal of a high-fat diet gives rise to a state of neuroimmune dysregulation, defined by an altered macrophage-driven inflammatory response together with heightened anxiety-like behavior and impaired motor performance.

Such weight cycles may facilitate not only renewed weight gain but also the persistence of inflammatory alterations even after the weight-loss period in the lean state, supporting the concept of an “inflammatory obesogenic memory”. Notably, this sustained inflammatory imprint can remain even although metabolic parameters and body weight appear normalized and may contribute to chronic anxiety-related manifestations.

These findings could contribute to a deeper understanding of the dangerous effects of weight cycling and may help guide future strategies aimed at mitigating inflammation-related effects not only to cardiovascular but also to mental-health complications associated with lifelong yo-yo dieting.

## Figures and Tables

**Table 1:** Diet composition and energy content of the standard diet (SAFE A04) and the high-fat diet (SAFE 260HF).

	SAFE^®^ A04	SAFE^®^ 260HF
Nitrogen Free Extract (carbohydrates)	60.4%	37.23%
Crude Protein	16.1%	19.84%
Crude Fat	3.1%	35.92%
Kcal/kg	3145	5428

**Table 2:** Body weight, food intake, and metabolic profile in Control and Weight Cycling Groups.

	Control Group	Weight Cycling Group	ES
Body weight (g)	27.6 ± 0.7	30.6 ± 1.6	0.76
Daily food intake (g)	3.7 ± 0.06	3.1 ± 0.1 ***	0.24
Fasting glucose (mg/dL)	232 ± 46.1	181.2 ± 20.1	0.73
Cholesterol (mg/dL)			
Total cholesterol	<100	<100	-
HDL-C	52 ± 2.9	52.3 ± 1.5	0.06
cLDL-C	33.1 ± 5.6	34.1 ± 2.8	0.11
Triglycerides (mg/dL)	<50	<50	-

Each value represents the mean ± SEM of the determinations obtained from 5 animals from Control Group and 7 animals from Weight Cycling Group. *** *p* < 0.001, compared to the Control Group value (Student’s *t*-test). HDL-C: high-density lipoprotein cholesterol; cLDL-C: calculated low-density lipoprotein cholesterol; ES: effect size.

**Table 3:** Effect of weight cycling on phagocytic percentage and activity of peritoneal macrophages.

	Control Group	Weight Cycling Group	ES
Phagocytic percentage (%)	49.9 ± 9.7	45.8 ± 7.1	1.05
Phagocytic activity (MFI)	47.3 ± 7.1	36.3 ± 7.6	0.67

Each value represents the mean ± SEM of the determinations obtained from 5 animals from Control Group and 7 animals from Weight Cycling Group. MFI: mean intensity fluorescence: ES: effect size.

**Table 4:** Effect of weight cycling on microbicide percentage and activity of peritoneal macrophages.

	Control Group	Weight Cycling Group	ES
Microbicide percentage (%)	62.9 ± 6.7	61.5 ± 7.8	0.08
Microbicide activity (MFI)	27.9 ± 3.4	27.1 ± 5.8	0.18

Each value represents the mean ± SEM of the determinations obtained from 5 animals from Control Group and 7 animals from Weight Cycling Group. MFI: mean intensity fluorescence; ES: effect size.

**Table 5:** Effect of weight cycling on the inflammatory profile of peritoneal macrophages.

Phenotypic Profile (% of Cells)		Control Group	Weight Cycling Group	ES
M1 (pro-inflammatory)	CD11c^+^	68.2 ± 6.1	81.2 ± 3.1 *	1.2
	iNOS^+^	2.2 ± 0.6	1.2 ± 0.4	0.92
	CD11c^+^ iNOS^+^	5.4 ± 1.2	5.1 ± 1.3	0.12
M2 (anti-inflammatory)	CD206^+^	7.8 ± 1.7	6.3 ± 1.3	0.3
	ARG-1^+^	10.4 ± 2.1	7.5 ± 1.2	0.74
	CD206^+^ ARG-1^+^	3.9 ± 0.6	3 ± 0.7	0.6
Ratios	CD11c^+^/CD206^+^	11.1 ± 2.9	12.3 ± 2	0.22
	iNOS^+^/ARG-1^+^	0.3 ± 0.1	0.2 ± 0.1	0.18

Each value represents the mean ± SEM of the determinations obtained from 5 animals from Control Group and 7 animals from Weight Cycling Group. * *p* < 0.05, compared to the Control Group value (Student’s *t*-test). ES: effect size.

**Table 6:** Effect of weight cycling on the number of infiltrated macrophages and the formation of crown-like structures (CLS) in WAT.

	Control Group	Weight Cycling Group	ES
Infiltrated macrophages (number)	21.3 ± 3	38.1 ± 3.5 **	1.99
CLS	0.4 ± 0.2	1.6 ± 0.4 *	1.24

Each value represents the mean ± SEM of the determinations obtained from 5 animals from Control Group and 7 animals from Weight Cycling Group, with 10 random fields analyzed per blinded sample. * *p* < 0.05, ** *p* < 0.01, compared to the Control Group value (Student’s *t*-test). ES: effect size.

## Data Availability

The original contributions presented in this study are included in the article. Further inquiries can be directed to the corresponding author.

## Abbreviations

The following abbreviations are used in this manuscript:

WHO	World Health Organization
CRP	C-reactive protein
WAT	White adipose tissue
SD	Standard diet
HFD	High-fat diet
EPM	Elevated plus maze
HDL-C	High-density lipoprotein cholesterol
cLDL-C	Calculated low-density lipoprotein cholesterol
TG	Triglycerides
PBS	Phosphate-buffered saline
OCT	Optimal cutting temperature
MFI	Mean fluorescence intensity
PFA	Paraformaldehyde
FITC	Fluorescein isothiocyanate
HE	Hydroethidine
CLS	Crown-like structures
SEM	Standard error of the mean
